# Sporadic Cryptosporidiosis, North Cumbria, England, 1996–2000

**DOI:** 10.3201/10.3201/eid1006.030325

**Published:** 2004-06

**Authors:** Stella Goh, Mark Reacher, David P. Casemore, Neville Q. Verlander, Rachel Chalmers, Margaret Knowles, Joy Williams, Keith Osborn, Sarah Richards

**Affiliations:** * Carlisle and District Primary Care Trust, Rosehill, Carlisle, United Kingdom;; † Communicable Disease Surveillance Centre, London, United Kingdom;; ‡ University of Wales, Aberystwyth, Ceredigion, Wales, United Kingdom;; § Singleton Hospital, Swansea, Wales, United Kingdom;; ¶ Cumberland Infirmary, Carlisle, United Kingdom;; # United Utilities, Great Sankey, Warrington, United Kingdom;; ** West Cumberland Hospital, Whitehaven, United Kingdom

**Keywords:** Cryptosporidium, Cryptosporidiosis, Sporadic, , Risk factors, Case-control study, Water, Water treatment , Public Water Supplies, Diarrhea, Immune suppressions

## Abstract

Risk factors for sporadic cryptosporidiosis were determined in 152 patients and 466 unmatched controls who resided in two local government districts in North Cumbria, North West England, from March 1, 1996, to February 29, 2000. Risk was associated with the usual daily volume of cold unboiled tap water drunk (odds ratio [OR] 1.40, 95% confidence intervals [CI] 1.14 to 1.71 per pint consumed per day [p = 0.001]) and short visits to farms (OR 2.02, 95% CI 1.04 to 3.90, p = 0.04). Fifty-six (84%) of 67 fecal specimens from patients obtained from January 1, 1998, and February 29, 2000, were *Cryptosporidium parvum* genotype 2 (animal and human strain). Livestock fecal pollution of water sources appears to be the leading cause of human sporadic cryptosporidiosis in this population and shows the need for better protection of water catchments from livestock and improved drinking water treatment in this area of England.

The protozoan parasite *Cryptosporidium parvum* is a leading cause of infectious diarrhea in humans and livestock with fecal-oral transmission by ingestion of oocysts (*1**,**2*). Infection is generally self-limiting, followed by variable protective immunity involving humoral and cell-mediated responses, except in the immune-suppressed, when infection may be prolonged and fatal (*2**,**3*). *Cryptosporidium* oocysts remain viable in water and damp soils for prolonged periods and are resistant to disinfectants at concentrations usually used in water treatment (*4**,**5*). Although sound, conventional water treatment is believed to substantially reduce the risk of viable oocysts passing into treated water, the possibility of low-level intermittent contamination has been recognized; whether such contamination affects public health is uncertain (*4**,**6*). Outbreak investigations have shown diverse modes of transmission, including contact with livestock (*7**,**8*); person-to-person transmission in households and care settings (*9*); consumption of contaminated foods and drinks, including milk (*1**,**10*); water from private supplies (*11*); and recreational water exposure (*12*). Infection may also be associated with travel to countries with higher incidence of cryptosporidiosis (*13*).

In 1992, a community outbreak of cryptosporidiosis occurred in residents of Allerdale and Copeland local government districts in North Cumbria, North West England, which compose part of the Lake District National Park; these areas have a predominantly agricultural and tourism-based economy and a population of approximately 160,000. The lakes have livestock farms and open grazing land abutting them. Approximately one third of the population received public water supplies from Ennerdale Lake, one third from Crummock Lake, and one third from a number of smaller sources. Water from Ennerdale and Crummock Lakes was disinfected with chlorine but unfiltered because the low level of particulate matter in these sources precluded chemically assisted flocculation. The smaller sources used for public supplies received a variety of conventional treatments, including coagulation, filtration, and chlorination, and chlorination alone. A small number of households had private supplies. A case-control study conducted during the outbreak showed a significant association between cryptosporidiosis and consuming cold unboiled mains tap water for persons served by water from Ennerdale Lake but no such association for those served by water from Crummock Lake and other water sources (North Cumbria Health Authority, Carlisle, 1992, unpub. data). After the outbreak, rates of laboratory-confirmed cryptosporidiosis from 1993 to 1995 were 31.2–44.2 per 100,000 in Allerdale and Copeland compared to 19.8 to 23.9 per 100,000 in the neighboring local government districts of Carlisle and Eden. Cases were not obviously clustered in time and could not be linked. A prospective case-control study was therefore undertaken to test the hypothesis that no dose-response relationship existed between consuming unboiled tap water from public water supplies and risk for sporadic *Cryptosporidium* infection in Allerdale and Copeland.

Medical and diagnostic microbiology services for Allerdale and Copeland residents were provided free at the point of use by the United Kingdom National Health Service and managed by North Cumbria Health Authority (*14*). The Authority was also responsible for maintaining the computer patient register and updating it for births, deaths, migration, and surveillance and control of infectious disease (*14*). The register held persons' name, sex, date of birth, home address, and postal (zip) code but not medical information. The postal codes were geographically referenced but did not share boundaries with public water supply distribution zones, government boundaries, or census enumeration districts.

**Methods**

Local ethical committees approved the protocol, and a study center was set up at North Cumbria Health Authority. All fecal specimens were examined for *Cryptosporidium* oocysts, regardless of whether this test was requested by the clinician. Family physicians and hospital clinicians were informed of the study, but not the main hypothesis, and were reminded to ensure best practice in investigating cases of diarrhea with the assistance of the three local microbiology laboratories. Laboratory staff was requested to immediately report *Cryptosporidium*-positive fecal smear results to the study coordinator by telephone.

**Epidemiology**

Five workers were trained to conduct the study in a standard manner by using written protocols and questionnaires. Both methods were pilot tested and their techniques refined before enrollment (*15*).

**Definitions**

Case-patients were defined as residents of Allerdale or Copeland with 1) diarrhea (three or more loose stools in a 24-hour period), 2) onset from March 1, 1996, to February 29, 2000, and 3) a fecal smear positive for *Cryptosporidium* oocysts but negative for other enteric pathogens. Patients were excluded if they had, within 14 days of onset of illness, contact with another household member with cryptosporidiosis or any diarrhea illness, traveled outside the United Kingdom, stayed away from home outside the study area within the United Kingdom for >7 nights, or if they or a household member had already been enrolled as a patient or control at any time during the study.

Controls were defined as residents of Allerdale or Copeland with no history of diarrhea (three or more loose stools in a 24-hour period) in the 14 days before interview. Potential controls were excluded if they had, within 14 days of interview, traveled outside the United Kingdom, stayed away from home outside the study area but within the United Kingdom for >7 nights, or if they or a household member had already been enrolled as a patient or control at any time during the study.

Three controls were randomly selected, by using a computer algorithm, from subtables of the health authority patient register with the same age span (0–5, 6–15, and 16+ years) and the same lead characters of the postal code as the case-patient. The process was repeated when necessary to replace persons who declined, could not be contacted, or met exclusion criteria. The patient register was compared with population estimates from the 2001 census.

**Interviews**

Interviews were conducted face-to-face at home. Participants <18 years of age were interviewed with parents or guardians, who acted as proxies for younger children, as appropriate. Informed consent was obtained and recorded. Case-patients and associated controls were enrolled as close to each other in time as possible.

For unboiled cold tap water, bottled water, and soft drinks, we asked about "usual" consumption without a time restriction. For all remaining exposures, including consuming pasteurized and unpasteurized milk, ice, and foods, exposure was sought for the 2 weeks before onset of illness for patients and before date of interview for controls.

The volume of different fluids drunk by study participants was determined by showing a standard picture card of a glass, cup, and mug; the volume was recorded according to the calibration on the card. The usual daily volume of water as cold unboiled tap water drunk at home was determined as water alone and as a diluent in cold fruit-squash type drinks. The usual daily volume of cold unboiled tap water drunk at work, school, or nursery at locations within Copeland and Allerdale District boundaries was determined the same way. The two usual daily volumes consumed at and away from home were added to give the total usual daily volume of cold unboiled tap water consumed within Copeland and Allerdale Districts. The usual daily volume consumed of bottled drinking water and soft drinks not diluted with water was determined separately without distinction between consuming it at home or away from home. Questions about consumption of ice in cold drinks were asked separately for ice made at home and ice consumed at work, school, or nursery.

The following types of contact were recorded: farms, farmed animals, and handling and feeding farm animals; slurry; household pets and feeding household pets; children's nurseries; and recreational exposure to water in swimming pools, rivers, and streams. Information about consumption and frequency of consumption of uncooked salad items, uncooked meat, uncooked sausage meat and sausages, yogurt, cheese, and cream was also elicited.

The nature of the water supply (public or private) and sewage services (public or septic tank) to the home were recorded and corroborated against water company records. Disruption to tap water or change in tap water color or taste in the week before onset of illness for patients, and in the week before date of interview for controls, was recorded. Information on sources, water treatment works, and the water that supplied each address's postal code was obtained from water company records and linked to individual patient and control records.

**Knowledge about *Cryptosporidium* Infection**

A series of television and newspaper articles on *Cryptosporidium* occurred in North Cumbria in April and May 1999. Study participants enrolled from April 28 to November 30, 1999, were also asked if they had heard of *Cryptosporidium* and what they knew about it.

**Data Entry and Analysis**

Data were double entered and differences edited and corrected in EpiInfo version 6.0.4.d. (Centers for Disease Control and Prevention, Atlanta, GA). Fluid intakes were analyzed as yes/no responses; within categories (<1/4 pint, 1/4–1 pint, >1–2 pints, and >2 pints) with χ^2^ tests for trend in single variable analysis; and as actual volume consumed in multivariable analysis (*16*).

Variables positively associated with infection at the p < 0.2 level in the single variable analysis, age, sex, and water supply zone were included in the initial multivariable model. Backward stepwise logistic regression was undertaken by comparing nested models using Likelihood Ratio Tests and GLIM software (*16**,**17*). Variables with significance p > 0.3 in iterations of the multivariable model were removed in stepwise fashion except for age, sex, and water supply zones, which were retained in all models regardless of their significance. Subsidiary analyses modeled usual daily consumption of tap water at home and usual total daily tap water consumption; cases and controls served by mixes of water from more than one source were omitted.

Microscopy of stained fecal smears for *Cryptosporidium* oocysts (*18*), *Giardia*, and culture for pathogenic enteric bacteria were undertaken by using standard methods at each of the three local microbiology laboratories. Smears in which *Cryptosporidium* oocysts were identified from January 1998 to February 2000 were also analyzed by polymerase chain reaction and restriction fragment length polymorphism typing of a region of the *Cryptosporidium* oocyst wall protein gene (*19**,**20*).

**Results**

None of the incident cases arising from the study population during the 4 years of the study were linked or clustered in time and space. All were considered sporadic infections eligible for inclusion in the study. No changes occurred in livestock farming, livestock densities, water sources, or water treatment within the study area during the study period.

**Potential Study Cases and Exclusions**

Two hundred seven case-patients were ascertained during the study period; 152 (73.4%) were enrolled, and 55 (26.6%) were excluded (Table 1). One refused to participate, one was unable to complete the interview, and two did not respond. Thirty-six (17.4%) were secondary to a laboratory-confirmed case in the household, 8 (3.9%) had traveled outside the United Kingdom, and 1 (0.5%) had traveled within the United Kingdom and stayed outside the study area >7 nights. Further single case-patients were excluded for having no history of diarrhea, mixed enteric infection, and being a visitor to, or resident outside, the study area. Two additional case-patients were excluded because a member of their household had already been interviewed as a case-patient or control earlier in the study.

**Table 1:** Recruitment and reasons for exclusion from study, United Kingdom (UK)

Exclusion criteria	No. (%) excluded
Case-patients (n = 207)	
Refusal to participate	1 (0.5)
Could not complete adequate interview	1 (0.5)
Did not respond to letters or phone calls	2 (1.0)
Not meeting the study case definition	
No history of diarrhea	1 (0.5)
Mixed enteric infection	1 (0.5)
Secondary case	36 (17.4)
Travel outside the UK in 14 days before onset	8 (3.9)
Travel in UK outside study area for >7 nights in the 14 days before onset	1 (0.5)
Visitor to study area	1 (0.5)
Residence outside study area	1 (0.5)
Case-patient or household member previously interviewed as case-patient or control	2(1.0)
Total potential cases excluded	55 (26.6)
Total cases enrolled	152 (73.4)
Controls (n = 778)	
Refusal to participate in interview	23 (3.0)
Unavailable at requested interview times	125 (16.1)
Said interview times were not convenient	35 (4.5)
Subtotal: refused, unavailable for interview	183 (23.5)
Address not found	3 (0.4)
History of diarrhea	46 (5.9)
Travel outside UK in the 14 days before interview	8 (1.0)
Travel in UK outside study area for >7 nights in the 14 days before interview	2 (0.3)
Not resident in study area in the 14 days before interview	3 (0.4)
Moved from study area	27 (3.5)
Resident outside study area	2 (0.3)
Control or household member already interviewed as a case-patient or control	7 (0.9)
Subtotal: did not meet study control definition	95 (12.2)
Interview cancelled by study team because three controls already enrolled for associated case	19 (2.4)
Interview cancelled by study team as potential control found to be in wrong age group	9 (1.2)
Reason for exclusion not recorded	3 (0.4)
Subtotal: not enrolled for administrative reasons or reason not recorded	31 (4.0)
Total potential controls excluded	312 (40.1)
Total controls enrolled	466 (59.9)

**Potential Controls and Exclusions**

Seven hundred seventy-eight potential controls were identified; 466 (59.9%) were enrolled, and 312 (40.1%) were excluded (Table 1). One hundred eighty-three (23.5%) refused to participate or were unavailable for interview. The address of three (0.4%) could not be found. Forty-six (5.9%) had a history of diarrhea, 8 (1.0%) had traveled outside the United Kingdom, and 2 (0.3%) had traveled within the United Kingdom away from the study area for >7 nights in the 2 weeks before interview. Twenty-seven (3.5%) had moved from the study area, 2 (0.3%) had an address outside the study area, and 7 (0.9%) shared a household with a patient or a control. Nineteen (2.4%) were not enrolled because 3 controls had already been recruited in association with the case, 9 (1.2%) were in the wrong age band, and 3 (0.4%) had no reason recorded.

**Study Population**

**Cases**

Of the 152 study case-patients, 86 (56.6%) were <6 years of age; 47 (30.9%) were 6–15; and 19 (12.5%) were >16 years. Eighty-two (53.9%) were male. More cases were detected in the first half of each year of the study (Figure). The average annual incidence rate was similar in populations served by water from Crummock Lake, Ennerdale Lake, and the other water sources combined (Table 2).

**Figure:**
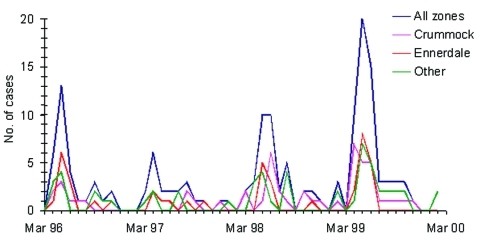
Case-patients recruited to the study by month of onset and water supply zone.

**Table 2:** Estimated average annual incidence of primary *Cryptosporidium* cases by water source, 1996–1999^a^

Water source	Population	Incidence (per 100,000 per y)
Crummock	58,295	24
Ennerdale	47,780	22
Crummock and Ennerdale	106,075	23.1
All other sources	59,699	22.6

^a^Population denominators from water company records.

In addition to diarrhea, the 152 patients reported abdominal pain (110 [72.4%]), vomiting (94 [61.8%]), fever (69 [45.4%]), anorexia (68 [44.7%]), and weight loss (56 [36.8%]). Thirty-seven patients were ill at interview. In 115 patients who had recovered when interviewed, the median duration of illness was 9 days (range 2–21). Nineteen (22.1%) of the 86 patients <5 years of age and 4 (8.5%) of 47 case-patients ages 6-15 years were admitted to hospital.

**Controls**

Three or four controls were recruited in association with 131 (86.2%) patients and one or two in association with the remainder. Patient and control groups were comparable by sex, local government district of residence, water sources and water supply zones, disruption and discoloration of tap water, nights spent away from home within the United Kingdom in the 2 weeks before onset or interview, and sewage services to the home (Table 3).

**Table 3:** Baseline characteristics of study population

Characteristics	Cases (%) 152 (100)	Controls (%) 466 (100)	p value
Sex			
M	82 (53.9)	236 (50.6)	0.539
F	70 (46.1)	230 (49.4)	
Age, y			
0–5	86 (56.6)	273 (58.6)	0.904
6–15	47 (30.9)	136 (29.2)	
16+	19 (12.5)	57 (12.2)	
Local government district			
Allerdale	83 (54.6)	248 (53.2)	0.838
Copeland	69 (45.4)	218 (46.8)	
Source of water/zone			
Crummock Lake/Crummock North	37 (24.3)	103 (22.1)	0.941
Crummock Lake/Crummock South	19 (12.5)	49 (10.5)	
Ennerdale Lake/Ennerdale North	29 (19.1)	101 (21.7)	
Ennerdale Lake/Ennerdale South	13 (8.6)	43 (9.2)	
Millom/Millom	19 (12.5)	54 (11.6)	
Quarry Hill/Quarry Hill	16 (10.5)	41 (8.8)	
Hausegill/Hausegill	3 (2.0)	5 (1.1)	
Hayknott/Hayknott	2 (1.3)	6 (1.3)	
Underscar/Underscar	1 (0.7)	3 (0.6)	
Fellside/Fellside	0	1 (0.2)	
Multiple sources/Mixed from >1 zone	11 (7.2)	51 (10.9)	
Different private water supplies	2 (1.3)	9 (1.9)	
Disruption of main supply in week before illness or outbreak?			
Y	8 (5.3)	14 (3.0)	0.294
N	143 (94.1)	448 (96.1)	
Not recorded	1 (0.7)	4 (0.9)	
Water discolored in the week before illness or outbreak?			
Y	14 (9.2)	33 (7.1)	0.398
N	130 (85.5)	431 (92.5)	
Not recorded	8 (5.3)	2 (0.4)	
Away from home in the 2 weeks before illness or outbreak?			
Y	49 (32.2)	163 (35.0)	0.603
N	103 (67.8)	303 (65.0)	
Sewage services to home			
Mains	130 (85.5)	411 (88.2)	
Septic tank	21 (13.8)	51 (10.9)	
Chemical toilet	1 (0.7)	0	
Not recorded	0	4 (0.9)	

**Time until Study Recruitment**

One hundred twenty-eight (84.2%) patients were interviewed within 1 week and 151 (99%) within 2 weeks of the date of the C*ryptosporidium*-positive fecal smear test report. The delay between reporting a case and enrolling the patient and associated controls was a median of 2.3 weeks (range 1–8).

**Knowledge about *Cryptosporidium***

Thirty six (75%) of 48 patients and 113 (67.7%) of 167 controls recruited from April 29 to November 30, 1999, stated that they had not heard of *Cryptosporidium* before being contacted for the study. The proportion without knowledge was similar for patients and controls recruited before and after July 8, 1999. Of the 66 persons who had previously heard of *Cryptosporidium*, 16 had knowledge of modes of transmission: 4 reported transmission could occur through water, drinks, or contact with farms and animals; 6 reported that transmission was by water only; and 6 reported transmission was by farm contact only.

**Single Variable Analysis**

Significant associations were seen with consuming cold unboiled tap water (odds ratio [OR] 2.12, 95% confidence interval [CI] 1.16 to 3.91, p = 0.012) with a significant dose-response relationship (χ^2^ test for trend p = 0.017). A significant dose-response relationship was also seen for the usual volume of cold unboiled tap water consumed at home (χ^2^ test for trend p = 0.005), but not for that consumed at the workplace, nursery, or school (χ^2^ test for trend p = 0.495) (Table A1). No association was found between consuming bottled water, ice, soft drinks, and pasteurized or unpasteurized milk. Consuming lettuce, tomatoes, mixed salad, and cream was associated with lower risk (p < 0.05).

Any contact with a farm was associated with a twofold increase in risk (OR 2.11, CI 1.4 to 3.2, p < 0.001). Risk was higher for short farm visits (OR 2.56, CI 1.57 to 4.17) and increased with the frequency of farm visits (χ^2^ test for trend p = 0.003) (Table A1). Risk was also increased by contact with farm animals (OR 2.23, CI 1.45 to 3.43), eating food within 2 hours of contact with farm animals (OR 3.11, CI 1.79 to 5.38), and stroking farm animals (OR 2.01, CI 1.17 to 3.42). Walking near slurry applied to fields was not associated with increased risk, but contact with slurry showed some evidence of increased risk (OR 2.0, CI 0.99 to 4.02).

Contact with pets at home or contact with pets with diarrhea did not increase risk. Risk was increased for feeding pets leftovers (OR 3.79, CI 1.0 to 14.69), with marginal evidence of risk for feeding pets raw vegetables (OR 2.09, CI 0.95 to 4.56) and biscuits (OR 1.76, CI 0.95 to 3.21). Contact with animals other than farm animals and home pets was not associated with infection (OR 0.84, CI 0.54 to 1.30). Risk was increased by having accidentally touched feces from any animal (OR 3.04, CI 1.33 to 6.94). Attendance at a playgroup or nursery and recreational exposure to water was not associated with infection (Table A1).

**Multivariable Analysis**

The usual volume of cold unboiled tap water consumed was independently associated with cryptosporidiosis (OR 1.40, CI 1.14 to 1.71 per pint consumed per day) in the final multivariable model (Table 4). A short visit to farms (OR 2.02, CI 1.04 to 3.9) was also significant. No difference in risk was found between the different water supply zones, irrespective of whether the zones received unfiltered water from Crummock and Ennerdale Lakes, other public supplies with a variety of conventionally filtered and unfiltered water, or private water supplies (Tables 3 and 4). Slight evidence was found for increased risk for feeding pets raw vegetables (OR 2.11, CI 0.98 to 4.56) and biscuits (OR 1.77, CI 0.94 to 3.35) but not for age, sex, consuming nonlocally produced cheese, contact with farms without cattle or sheep, or with cattle (Table 4). Subsidiary analysis showed that usual volume of cold unboiled tap water consumed at home was also a significant risk factor. A further analysis that excluded persons whose house was served by a mixed public water supply found similar results (not shown).

**Table 4:** Final multivariable modela

Variables	Cases	Controls	Adjusted OR	CI	p value
Total	152	466			
Sex					
M	82	236	1.1	0.73 to 1.67	0.64
F	70	230	1		
Age			0.99/y	0.97 to 1.01	0.29
Water supply zones					
Crummock North	37	103	1		0.71
Crummock South	19	49	1.28	0.61 to 2.67	
Ennerdale North	29	101	0.95	0.51 to 1.78	
Ennerdale South	13	43	0.9	0.39 to 2.09	
Millom	19	54	1.04	0.51 to 2.13	
Quarry Hill	16	41	1.36	0.61 to 3.05	
Hausegill	3	5	1.37	0.26 to 7.11	
Hayknott	2	6	0.89	0.16 to 4.85	
Underscar	1	3	1.83	0.16 to 20.37	
Fellside	0	1	0.002	0 to ∞	
Mixed from >1 zone	11	51	0.77	0.34 to 1.77	
Private water supplies	2	9	0.004	0 to 203.4	
Usual daily volume of cold unboiled tap water drunk^b^			1.40/pint	1.14 to 1.71	0.001
Any short visit to a farm					
Y	40	57	2.02	1.04 to 3.9	0.04
N	107	391	1		
Fed pet raw vegetables					
Y	13	20	2.11	0.98 to 4.56	0.06
N	139	446	1		
Fed pet biscuits					
Y	21	39	1.77	0.94 to 3.33	0.08
N	131	427	1		
Ate nonlocally produced cheese					
Y	113	322	1.49	0.91 to 2.43	0.1
N	34	139	1		
Contact with farms without cattle or sheep					
Y	15	19	1.96	0.79 to 4.88	0.15
N	125	417	1		
Contact with a cattle farm					
Y	15	29	1.67	0.73 to 3.82	0.23
N	125	407	1		

^a^OR, odds ratio; CI, confidence interval. ^b^At home and away from home in the Allerdale/Copeland area.

**Genotyping Results**

Genotyping was undertaken in 67 of 101 cases from 1998 to 2000. All the smears were confirmed positive, and 56 (83.6%) of tested smears were *C. parvum* genotype 2 (animal and human) strain, 1 (1.5%) was genotype 1 (human strain), and 10 (14.9%) could not be typed.

The primary care patient register was reviewed after patients were recruited for the study. The register contained 166,376 names of Allerdale and Copeland residents compared to a population of 162,809 enumerated at the 2001 census (available from www.statistics.gov.uk/census2001). The computer algorithm used to randomly select potential controls generated 125 tables of registered patients' names, where registered patients were within the same age category as study case-patients and had the same lead characters of the postal code of residence as study case-patients. The tables contained a median of 496 (range 9–7,800) names.

**Discussion**

Drinking cold unboiled tap water from public drinking water supplies was a highly significant risk factor for sporadic human cryptosporidiosis, regardless of the water source. To our knowledge, this study is the first to show that drinking from public water supplies is an important risk factor for sporadic human *Cryptosporidium* infection. Most cases were in children, consistent with previous reports from England and Canada (*21**,**22*). Many patients required admission to hospital, showing the seriousness of illness. Infection in study patients was also associated with short visits to farms and predominantly with the *C. parvum* genotype 2 (animal and human) strain, consistent with farmed livestock's being a major source of infection. Risk was not increased by contact with pets.

Excluding study participants by recent travel ensured that environmental exposures most likely occurred within the study area. Excluding household contact with an earlier onset case ensured that person-to-person transmission within the household was unlikely to have occurred in study case-patients.

The number of patients who sought medical attention was likely to have been high because the National Health Service provided free medical care within the study area at the point of use and because all fecal specimens were tested for *Cryptosporidium* by National Health Service laboratories without charge. Although only half of patients with cryptosporidiosis may seek medical attention in the United Kingdom (*23*), we have no reason to suppose that risk factors for patients who do not visit healthcare facilities would differ substantially from those that did.

Matching refers to pairing cases with one or more controls on the basis of their similarity in selected variables, with the objective of eliminating bias (*24**,**25*). We undertook stratified random sampling from a population list to select potential controls and adjusted by using multiple regression analysis, which is one of a number of alternative designs to matching (*24**,**25*).

Refusal to participate was low at 4 (1.9%) of 205 cases and 183 (23.5%) of 778 potential controls. Lower response rates in controls compared to cases is expected (*24*). Care was taken during the design and conduct of the study to mask interviewers to the tap water hypothesis. Interviewer training emphasized that all risk factors were plausible and required equal care in measurement. A survey after media coverage indicated little knowledge about risk factors for cryptosporidiosis by patients or controls. Patients may have increased fluid consumption after the onset of illness. Study participants were therefore asked to report their "usual consumption" of unboiled tap water, bottled water, and soft drinks, without a time restriction. Although a bias towards a patient's recalling consumption of fluids after onset of illness could explain some of the association with tap water that we observed, we do not think it can explain it entirely. In particular, fluid volumes were measured in the same way for unboiled tap water, bottled water, and soft drinks, but the highly significant association and dose-response relationship were observed only for unboiled tap water. An interview date bias was avoided because patients and associated controls were enrolled within a short time of each other.

Cryptosporidiosis in HIV-infected persons is associated with consuming unboiled tap water. We do not believe that undetected HIV infection or other causes of immune suppression could have been a major confounding factor. Only two new cases of HIV infection would be expected in Allerdale and Copeland each year even if rates for the whole North West of England were applied to the study population. However, our findings reinforce the need for immune-compromised persons, including those with HIV infection, to avoid drinking unboiled tap water (*26**,**27*). Allerdale and Copeland had very similar levels of social deprivation (*28*). Moreover, controls resided in approximately the same locality as patients, as defined by shared lead characters of the postal code. Therefore, a systematic difference in social deprivation between patients and controls was unlikely and would not have explained the associations we observed.

The spring peak and smaller autumn peak in our cases are consistent with previous reports from England and Wales. These peaks are attributed to lambing, calving, and runoff from spring rains and to summer travel to countries with higher incidence of cryptosporidiosis (*4**,**13*).

Our findings contrast with a case-control study in Adelaide and Melbourne, which did not detect increased risk for sporadic cryptosporidiosis associated with the public water supplies (*29*). This difference may reflect the quality of the source waters, of water treatment, or both in these cities. Swimming pool exposure was the most significant risk factor in the Australian study. Regularly consuming raw vegetables was protective in that study. We also observed a protective association with lettuce, tomatoes, and mixed salad, and additionally for cream, in single variable analysis. These foods might have conferred a direct protective nutrient effect, been markers for more favorable general nutrition, or contained small numbers of oocysts derived from water used for irrigation and preparation sufficient to induce immune boosting (*3**,**30**,**31*). A recent study of sporadic cryptosporidiosis in the San Francisco Bay Area also failed to show an association with tap water, but the study was small (*32*).

Most of our patients were children, which suggests that older members of our study population were mainly immune, probably because of long-term immune boosting from low-level intermittent contamination of water supplies and contact with livestock (*31**,**33*). This observation is consistent with recent seroprevalence studies in blood donors resident in midwestern American cities and indicates lower seroprevalence of *Cryptosporidium* antibodies in populations served by deep borehole water compared to lake and river water supplies (*34**,**35*).

No association was seen for contact with pets at home, pets with diarrhea, or feeding tinned pet meat, raw meat, or pellets. However, feeding pets biscuits and raw vegetables was associated with slightly increased risk in single variable analysis and in the final multivariable model. These food types may be markers for more intimate contact with animal secretions; feeding raw vegetables may indicate contact with contaminated water in preparation. Although contamination of pet biscuits is possible, these products are dry and manufactured at high temperature; thus, survival of oocysts within these foods seems unlikely. Accidental hand contact with the feces of any type of animal was significant in single variable analysis but not in the final multivariable model. These results suggest that sound hygiene in cleaning animal feeding utensils, avoiding cross-contamination between pet and human food preparation areas, and good hand hygiene are desirable but that pets and pet feeding were not major risk factors for cryptosporidiosis in this population.

The findings of this study are consistent with the decline in human *Cryptosporidium* infection throughout England and Wales, coincident with the foot and mouth disease epidemic in livestock during 2001 (*13*). These two facts strongly suggest that livestock reservoirs of *Cryptosporidium* contribute substantially to sporadic human cryptosporidiosis in North Cumbria and in England and Wales as a whole, through low-level intermittent *Cryptosporidium* oocyst contamination of public drinking water supplies. Our results support the need for rigorous risk assessment of water sources and, where indicated, improved catchment control. Our results are also in accord with recent U.K. legislation that requires continuous monitoring of *Cryptosporidium* oocyst concentrations in treated water from at-risk supplies (*36*). Advanced methods of filtration, disinfection, and UV light treatment may be required to further decrease the risk for cryptosporidiosis from public water supplies (*5**,**37**,**38*).

The water company installed membrane filtration during 2000 at works served by Crummock and Ennerdale Lakes, which previously provided chlorination alone. The impact of this intervention will be presented in a separate article.

## Appendix

**Table A1:** Single variable analysis^a^

Water and fluid consumption	Cases	Controls	OR	LCL	UCL	p value
Usually drink cold tap water without boiling it first						
Y	136	373	2.12	1.16	3.91	0.012
N	16	93	1			
Usual daily consumption of cold unboiled tap water at home (pints)						
<¼	23	106	1			0.005^b^
¼–1	65	218	1.37	0.79	2.42	
>1–2	40	96	1.92	1.03	3.59	
>2	21	40	2.42	1.14	5.13	
Usual consumption of cold, unboiled tap water away from home (pints) at work/nursery/school in the Copeland/Allerdale area						
<¼	83	273	1			0.495^b^
¼–1	39	110	1.17	0.73	1.85	
>1	2	5	1.32	0.17	7.82	
Was usual consumption unboiled tap water away from home/at work/nursery outside the Copeland/Allerdale area?						
Y	13	31	1.29	0.62	2.67	0.573
N	137	422	1			
Usual daily volume of cold, unboiled tap water drunk (pints) at home and away from home in the Allerdale and Copeland area						
<¼	19	108	1			0.017^b^
¼–1	58	175	1.88	1.03	3.47	
>1–2	45	121	2.11	1.12	4.01	
>2	28	62	2.57	1.26	5.24	
Do you usually drink bottled water?						
Y	16	36	1.43	0.73	2.79	0.323
N	133	429	1			
Do you usually drink soft drinks?						
Y	87	283	0.86	0.58	1.27	0.467
N	64	178	1			
If yes, how much soft drinks, on average, do you drink per day (pints)						
<¼	66	181	1			0.358^b^
¼–¾	36	110	0.9	0.55	1.47	
1–2	31	103	0.83	0.49	1.39	
>2	11	40	0.75	0.34	1.63	
Did you have any drinks with ice added: (cases) in the 2 weeks before illness? (controls) in the 2 weeks before interview?						
Y	29	106	0.83	0.51	1.36	0.514
N	116	354	1			
If yes, consumption of ice at home: (cases) in the 2 weeks before illness? (controls) in the 2 weeks before interview?						
Y	11	57	0.56	0.27	1.15	0.119
N	141	409	1			
Drink pasteurized milk						
Y	134	396	1.3	0.72	2.36	0.431
N	18	69	1			
Pints of pasteurized milk on average per day (pints)						
<¼	19	71	1			0.221^b^
¼–¾	66	153	1.61	0.87	3.01	
>1	56	202	1.04	0.56	1.94	
Drink unpasteurized milk						
Y	9	22	1.28	0.53	3.02	0.699
N	142	443	1			
Food consumption	Cases	Controls	OR	LCL	UCL	p value
Lettuce						
Y	36	166	0.58	0.37	0.9	0.013
N	112	298	1			
Tomatoes						
Y	42	170	0.65	0.43	1	0.048
N	110	291	1			
Coleslaw						
Y	26	93	0.85	0.51	1.41	0.572
N	122	369	1			
Mixed salad						
Y	25	123	0.56	0.33	0.92	0.020
N	124	339	1			
Raw sausages						
Y	6	12	1.56	0.51	4.61	0.4062
N	145	452	1			
Other raw meat						
Y	8	13	1.93	0.71	5.14	0.233
Fresh raw vegetables						
Y	47	177	0.76	0.5	1.16	0.216
N	100	288	1			
Local cheese						
Y	5	8	1.94	0.54	6.71	0.3252
N	144	446	1			
Other cheese						
Y	113	322	1.43	0.91	2.27	0.124
N	34	139	1			
Yogurt						
Y	113	346	1.03	0.66	1.63	0.968
N	37	117	1			
Ice cream						
Y	98	338	0.68	0.45	1.04	0.072
N	53	125	1			
Cream						
Y	18	95	0.53	0.3	0.95	0.029
N	131	369	1			
Farm and farm animal contact	Cases	Controls	OR	LCL	UCL	p value
Did you have contact with farms?						
Y	59	108	2.11	1.4	3.2	<0.001
N	91	352	1			
Any short visit at a farm						
Y	40	57	2.56	1.57	4.17	<0.001
N	107	391	1			
Frequency of farm visit						
0	91	352	1			0.003^b^
1–5	44	82	2.08	1.32	3.27	
6–10	3	2	5.8	0.78	50.38	
>11	8	11	2.81	1	7.81	
Any contact with a dairy farm						
Y	13	18	2.38	1.06	5.31	0.033
N	127	418	1			
Any contact with a farm with cattle or sheep						
Y	15	29	1.68	0.83	3.4	0.164
N	125	407	1			
Any contact with a farm with cattle and sheep						
Y	10	20	1.6	0.67	3.73	0.334
N	130	416	1			
Any contact with a farm without cattle or sheep						
Y	15	19	2.63	1.22	5.66	0.010
N	125	417	1			
Any overnight stay at a farm						
Y	1	5	0.61	0.03	5.45	1.000
N	146	443	1			
Did you have contact with farm animals?						
Y	53	92	2.23	1.45	3.43	<0.001
N	94	364	1			
Frequency of farm animal contact						
0	94	364	1			
1 to 20	38	68	2.16	1.34	3.5	0.051
>20	4	5	3.1	0.68	13.6	
Eat food within 1 to 2 hours of contact						
Y	32	41	3.11	1.79	5.38	<0.001
N	96	382	1			
Did you feed farm animals by hand?						
Y	23	45	1.74	0.97	3.12	0.061
N	116	396	1			
Stroke any farm animal						
Y	29	49	2.01	1.17	3.42	0.009
N	123	417	1			
Any other contact with a farm animal						
Y	28	35	2.78	1.57	4.93	<0.001
N	124	431	1			
Did you have any contact with fields recently sprayed with slurry						
Y	5	10	1.72	0.5	5.64	0.351
N	123	422	1			
Walk close to slurry in a field						
Y	6	9	2.31	0.71	7.34	0.122
N	122	423	1			
Any contact with slurry						
Y	16	28	2	0.99	4.02	0.052
N	116	406	1			
Contact with pet animals at home						
Y	98	293	1.08	0.72	1.62	0.77
N	53	171	1			
Contact with pets with diarrhea						
Y	6	23	0.78	0.27	2.12	0.763
N	85	254	1			
Feed your pet raw meat						
Y	0	2	0	0	12.74	1.000
N	152	464	1			
Feed your pet tinned meat						
Y	42	126	1.03	0.67	1.59	0.97
N	110	340	1			
Feed your pet pellets						
Y	2	6	1.02	0	5.73	1.000
N	150	460	1			
Feed your pet biscuits						
Y	21	39	1.76	0.95	3.21	0.070
N	131	427	1			
Feed your pet leftovers						
Y	6	5	3.79	1	14.69	0.031
N	146	461	1			
Feed your pet raw vegetables						
Y	13	20	2.09	0.95	4.56	0.069
N	139	446				
Feed your pet other food						
Y	44	127	1.09	0.71	1.67	0.763
N	108	339	1			
Did you have contact with any other animals or birds						
Y	75	248	0.84	0.54	1.30	0.475
N	51	142	1			
Did you or your child accidentally touch any dirt or feces from any animal						
Y	13	17	3.04	1.33	6.94	0.006
N	89	354	1			
Play groups and recreational water exposure	Cases	Controls	OR	LCL	UCL	p value
Contact with play group						
Y	61	164	1.27	0.84	1.9	0.269
N	82	279	1			
Swim at a swimming pool						
Y	44	141	0.94	0.61	1.44	0.838
N	108	325	1			
Engage in water sports						
Y	2	18	0.33	0.05	1.53	0.1852
N	150	448	1			
Go paddling						
Y	22	57	1.23	0.7	2.17	0.531
N	128	408	1			
Any other contact with fresh water						
Y	16	39	1.29	0.66	2.49	0.516
N	133	418	1			

^a^OR, odds ratio; LCL, lower confidence limit; UCL, upper confidence limit. ^b^χ^2^ test for trend.
